# A soluble tau fragment generated by caspase-2 is associated with dementia in Lewy body disease

**DOI:** 10.1186/s40478-019-0765-8

**Published:** 2019-07-30

**Authors:** Benjamin R. Smith, Kathryn M. Nelson, Lisa J. Kemper, Kailee Leinonen-Wright, Ashley Petersen, C. Dirk Keene, Karen H. Ashe

**Affiliations:** 1 N. Bud Grossman Center for Memory Research and Care, University of Minnesota, 2101 6th Street SE, Minneapolis, 55455 USA; 2 Department of Neurology, University of Minnesota, Minneapolis, USA; 3 Department of Medicinal Chemistry, University of Minnesota, Minneapolis, USA; 4 Division of Biostatistics, School of Public Health, University of Minnesota, Minneapolis, USA; 5 Department of Pathology, University of Washington, Seattle, 98195 USA; 6 Minneapolis VA Medical Center, Minneapolis, 55417 USA

**Keywords:** Lewy body, Dementia, Parkinson’s, Caspase-2, Tau

## Abstract

Lewy body diseases are neurodegenerative disorders characterized by Lewy bodies in the brain. Lewy body dementia (LBD) refers to two forms of Lewy body disease: Parkinson’s disease with dementia (PDD) and dementia with Lewy bodies (DLB). Tau is a cytoskeletal protein found in neurofibrillary tangles, but not Lewy bodies. The gene encoding tau, MAPT, is a well-established genetic risk factor for LBD; odds ratios of the H1:H2 MAPT haplotypes have been reported in the range of 2 to 4. Despite this genetic association, the mechanism by which tau contributes to dementia is unclear. Recently, a soluble form of tau, Δtau314, which is generated when caspase-2 (Casp2) cleaves tau at Asp314, was reported to be associated with impaired cognition in mice modeling frontotemporal dementia, and with mild cognitive impairment and Alzheimer’s disease (AD) in humans. To investigate whether Δtau314 is associated with dementia in Lewy body disease, we compared Δtau314 levels in aqueous extracts from the superior temporal gyrus of pathologically confirmed LBD (*n* = 21) and non-dementia Parkinson’s disease (PD) (*n* = 12). We excluded subjects with AD or microvascular pathology, which could mask potential associations of Δtau314 with LBD.

Using a Δtau314-specific ELISA, we found ~ 2-fold higher levels of Δtau314 in LBD relative to PD (*p* = 0.009). Additionally, we found ~40% lower levels of soluble total tau and the neuronal marker β-III-tubulin in LBD. These results suggest that in LBD, there is substantial neuron loss or axonal degeneration in the neocortex but disproportionately high levels of Δtau314 in the surviving neurons.

Our results indicate an association between Δtau314 and dementia in Lewy body disease. Cleavage of tau by Casp2 promotes the mislocalization of tau to dendritic spines leading to a reduction in postsynaptic AMPA receptors and excitatory neurotransmission, which suggests a mechanism of the synaptic dysfunction underlying cognitive impairment in LBD. These findings support the potential of Casp2 as a novel drug target for treating LBD.

## Introduction

Dementia is a frequent feature of Lewy body disease, a class of neurodegenerative conditions characterized by the presence of Lewy bodies in the brain. Lewy bodies are cytoplasmic inclusions containing α-synuclein proteins. Lewy body dementia (LBD) refers to demented patients who have Lewy body pathology. The two forms of LBD —Parkinson’s disease with dementia (PDD) and dementia with Lewy bodies (DLB)—differ in clinical presentation. Dementia appears before or within 1 year of motor signs in DLB, while motor signs appear 1 year before dementia in PDD. There is a strong but imperfect association between neocortical Lewy bodies and dementia. Neocortical Lewy bodies were found in 49.5% of patients with PDD and 15.2% of patients with Parkinson’s disease without dementia (PD) [10].

Tau is a microtubule-associated protein encoded by the MAPT gene on Chr17q21.31. There are two major alleles of Chr17q21.31, termed H1 and H2. The H2 haplotype arose through the inversion of a ~ 900 Kb segment of Chr17q21.31, which spans several genes, including MAPT. Many GWAS studies of LBD show a protective effect of the H2 allele. The H1:H2 odds ratio (OR) for PDD versus PD was reported to be 3.7 [16]. In PD, homozygosity for H1 was associated with accelerated progression to dementia [7]. The H1:H2 OR for DLB versus controls was reported to range from 2.3–3.3 [12]. However, in a larger study [8] MAPT showed only nominal association.

Tau is an axonal protein whose normal function includes promoting the polymerization of tubulin to form microtubules in axons. Under disease conditions, tau redistributes to the soma and dendrites, and insoluble tau accumulates in cytoplasmic neurofibrillary tangles. Using tau positron tomography to image neurofibrillary pathology in humans, increased cortical uptake was found in LBD relative to normal control subjects [6].

Soluble forms of tau also play pathogenic roles. A soluble tau fragment, Δtau314, forms when caspase-2 (Casp2) cleaves tau at Asp314 [23]. Rendering tau resistant to Casp2 cleavage preserves memory function, and lowering Casp2 restores memory function in mice expressing mutant human tau linked to frontotemporal dementia [23]. Cleavage and phosphorylation of tau lead to tau mislocalization to dendritic spines, reduced AMPA receptor levels in the postsynaptic membrane, and abnormal excitatory neurotransmission [9, 23]. Cells lacking Casp2 or expressing Casp2-resistant tau do not show tau mislocalization or impaired synaptic transmission [23].

Casp2 is a member of the family of cysteine aspartic proteases, of which there are 12 in humans. Casp2 has both apoptotic and non-apoptotic roles, the latter including the modulation of autophagy and cell cycle arrest. Cleavage of tau to generate Δtau314 is another non-apoptotic effect of Casp2.

The levels of Δtau314 were ~ 3-fold higher in brain extracts from patients with mild cognitive impairment (MCI) and Alzheimer’s disease (AD) relative to cognitively intact subjects [23], suggesting that Δtau314 may be a biomarker for impaired cognition. The relationship between Δtau314 and cognition has never been examined in Lewy body disease. Here we tested the hypothesis that Δtau314 is associated with dementia in Lewy body disease, independent of AD or microvascular pathology.

## Materials and methods

### Aim, design and setting of the study

The aim of the study was to compare levels of Δtau314 in patients with Lewy body disease with (i.e., LBD) and without dementia (i.e., PD), in the absence of AD or microvascular pathology. We obtained frozen brain specimens from the superior temporal gyrus of donated brains from research participants in the Pacific Udall Center, the Alzheimer’s Disease Research Center, and the Adult Changes in Thought Study from the University of Washington Neuropathology Core. At autopsy, every brain underwent comprehensive neuropathological evaluation per the latest diagnostic guidelines for the assessment of Alzheimer’s disease neuropathologic change, Lewy body disease, vascular brain injury, and other disorders. Lewy body disease specimens were selected using the following criteria: presence of Lewy bodies in the brainstem, limbic area, or neocortex; absence of a “moderate” or “frequent” CERAD neuritic amyloid plaque score; and few or no microvascular lesions. A clinical diagnosis of dementia was assigned based on psychometric testing and formal review at consensus conferences specific to each study. A total of 21 LBD and 12 PD specimens met these criteria.

### Demographics and clinical characteristics of the subject population

There was no significant difference in age (PD = 82.8; LBD = 77.3), sex (PD = 58% male; LBD = 81% male), PMI (PD = 5.5; LBD = 6.3), brain weight (PD = 1231 g; LBD = 1339 g), or months since last evaluation (PD = 31.3; LBD = 45.8) between the groups (Table 1).

**Table 1:** Demographic and clinical characteristics of subject population

	PD	PDD	Total	*p* value
Age (years)				
Group size	12	21	33	
Mean ± SD	82.8 ± 10.5	77.3 ± 13.1	79.3 ± 12.4	
Range	57–98	42–96	42–98	0.19^a^
Sex				
Group size	12	21	33	
Male	7	17	24	
Female	5	4	9	0.23^b^
PMI (h)				
Group size*	11	21	32	
Mean ± SD	5.5 ± 2.2	6.3 ± 4.0	6.0 ± 3.5	
Range	1.9–11	2–16.3	1.9–16.3	0.45^a^
Brain weight (g)				
Group size*	11	17	28	
Mean ± SD	1231 ± 131	1339 ± 236	1296 ± 205	
Range	1010–1450	1070–2017	1010–2017	0.14^a^
Last evaluation (months)				
Group size	8	14	22	
Mean ± SD	31.3 ± 35.4	45.8 ± 29.1	40.5 ± 31.5	
Range	15.3–118.5	10.2–98.6	10.2–118.5	0.34^a^

^a^Unpaired t-test with Welch’s correction

^b^Fisher’s exact test

*Data for one or more patient was unavailable

### Pathological characteristics of the subject population

All subjects had Lewy body disease. The distribution of Lewy bodies in the brain in the PD and LBD groups differed significantly (Table 2). All subjects had sparse or no neuritic amyloid plaques and Braak Stages ≤ 4. There were no significant differences in Braak Stages or CERAD scores between the LBD and PD groups (Table 2).

**Table 2:** Pathological characteristics of subject population

	PD	PDD	Total	*p* value
Lewy bodies				
Group size	12	21	33	
Neocortical	3 (25%)	18 (86%)	21 (64%)	
Limbic	3 (35%)	0 (0%)	3 (9%)	
Brainstem	6 (50%)	3 (14%)	9 (27%)	0.001^a^
Group size*	11	20	31	
Braak 0	0 (0%)	2 (10%)	2 (6%)	
Braak 1	3 (27%)	5 (25%)	8 (26%)	
Braak 2	3 (27%)	2 (10%)	5 (16%)	
Braak 3	3 (27%)	6 (30%)	9 (29%)	
Braak 4	2 (18%)	5 (25%)	7 (23%)	
Braak 5	0 (0%)	0 (0%)	0 (0%)	
Braak 6	0 (0%)	0 (0%)	0 (0%)	0.86^a^
CERAD				
Group size*	12	20	32	
None	1 (8%)	8 (40%)	9 (28%)	
Sparse	11 (92%)	12 (60%)	23 (72%)	0.10^b^

^a^Chi-squared test

^b^Fisher’s exact test

*Data for one or more patient was unavailable

### Biochemical extraction

Superior temporal gyrus was collected at the University of Washington fresh during rapid autopsies, flash frozen in liquid nitrogen, and stored at -80C until the samples were shipped on dry ice to the University of Minnesota. Never-thawed superior temporal gyrus samples were extracted in Tris-buffered saline (TBS) containing 50 mM Tris-HCl (pH 7.4), 150 mM NaCl, protease inhibitor cocktail, 1 mM phenylmethylsulfonyl fluoride, 1 mM phenanthroline monohydrate and phosphatase inhibitors. Homogenates were centrifuged at 16,000 g for 60 min to pellet nuclei and large debris, and the supernatant was collected.

### APOE genotyping

Human brain tissue was homogenized and DNA was extracted using Qiagen DNeasy Blood and Tissue Kit (Cat 69506). DNA was quantified using Thermo Scientific NanoDrop spectrophotometer. APOE status was determined using APOE c.388 T > C and APOE c.526C > T Novallele Genotyping Assays (Canon BioMedical Inc. Cat40394 and Cat 40395) run on a Roche Lightcycler 480 system.

### Δtau314 and Total tau measurements by enzyme-linked Sandwich immunoassay

Samples were analyzed using Quanterix™ ultra-sensitive, single molecule array (Simoa) technology. 4F3 monoclonal antibodies were custom-generated by Precision Antibodies™. Δtau314 measurements were performed using 4F3-coated magnetic beads for protein capture and a combination of biotinylated BT2/HT7 antibodies for detection. Total tau (T-tau) was measured using Tau 2.0 kits (Quanterix™). All measurements were performed in triplicate on the same day in the same run using the same reagents, and experimenters were blind to clinical status.

### β-III-tubulin and GAPDH measurements by Western blotting

TBS extracts were normalized to protein levels (BCA assay, Thermo Fisher), diluted in reducing sample buffer, size-fractionated by SDS-PAGE on 10% Tris-HCl gels (Bio-Rad), and transferred onto 0.2 μm nitrocellulose membranes. Immunoblots were probed with β-III-tubulin (Sigma Aldrich) and GAPDH (Cell Signaling) antibodies and visualized using chemiluminescence reagents (Pierce) followed by exposure onto autoradiography film (Gene Mate). Band densities were measured using Fiji software (NIH). TBS extracts for Casp2 detection were run on 10.5–14% gradient gels (Bio-Rad) and visualized using a Casp2 specific antibody (Abcam).

### Statistical analysis

Groups were compared using the Mann-Whitney test or Student’s two-tailed t-tests with Welch’s correction. In addition, a multiple linear regression model was fitted with the logarithm of measured values (Δtau314, total tau (T-tau), or Δtau314: T- tau) and age, sex, and PMI. *P*-values for the comparisons are presented when using a two-sample t-test on the logged measure, which is equivalent to the linear regression with no adjustment variables. Demographics were analyzed using unpaired t-test with Welch’s correction or Fisher’s exact test. Pathological characteristics were analyzed using χ-squared or Fisher’s exact tests. AUC’s were compared using DeLong’s test for correlated ROC curves.

## Results

### Soluble Δtau314 is higher in Lewy body dementia (LBD) than non-dementia Parkinson’s disease (PD)

**Fig. 1:**
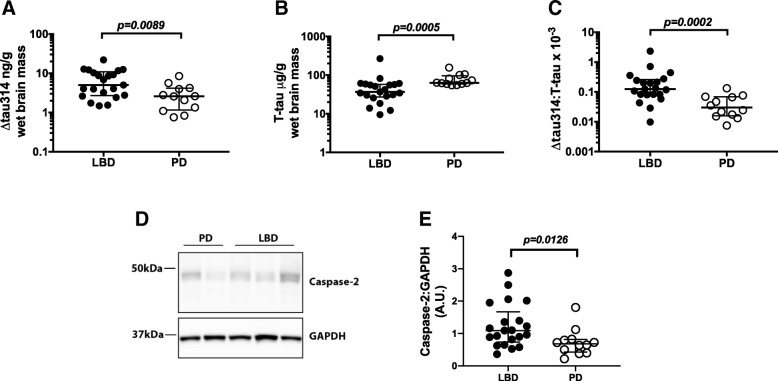
∆tau314 and caspase-2 levels are higher in LBD than PD. **a-c** Quantification of Δtau314 and T-tau in aqueous extracts from the superior temporal gyrus. **a** ∆tau314 is higher in LBD than PD. **b** T-tau is lower in LBD than PD. **c** The ∆tau314: T-tau ratio is higher in LBD than PD. **d** Representative western blots of Casp2 and GAPDH in aqueous extracts. **e** Quantification of Casp2 normalized to GAPDH. Casp2 levels are higher in LBD than PD. Data were analyzed using Mann-Whitney tests. Bars indicate the medians and interquartile ranges

We tested the hypothesis that Δtau314 is a biomarker for dementia in Lewy body disease. We measured Δtau314 in aqueous extracts from the superior temporal gyrus of 12 PD and 21 LBD patients, and found ~ 2-fold higher levels of Δtau314 in LBD (5.035; 2.696–10.95 ng/g) than PD (2.59; 1.16–4.163 ng/g) (Fig. 1a). (Note: In this paper, all tau measurements are presented as the amount of the protein of interest relative to wet brain mass, and the numerical values represent median; first - third interquartile intervals.) Applying either a Mann-Whitney test, two-tailed t-test with Welch’s correction, or multiple regression model adjusted for age, sex, and PMI, we found that the levels of Δtau314 were significantly higher in LBD than PD (Table 3).

**Table 3:** Statistics for Fig. 1. Data were analyzed using two-tailed unpaired non-parametric Mann-Whitney test, two-tailed t-test, and multiple-regression model adjusted for age, sex, and post-mortem interval

	Mann-Whitney test	Two-sample t-test	Multiple linear regression (adjusted for age, sex, PMI)
Δtau314	*p* = 0.0089	*p* = 0.0075	*p* = 0.0229
Total tau	*p* = 0.00049	*p* = 0.0012	*p* = 0.0090
Δtau314/Total tau	*p* = 0.00024	*p* = 0.00026	*p* = 0.0020

### Caspase-2 is higher in LBD than PD

Casp2 is produced as a zymogen containing a caspase activation and recruitment domain (CARD) situated in the amino-terminus of the protease domain. Like other caspases, proteolytic removal of the CARD activates Casp2. However, oligomerization of the intact zymogen is also sufficient to activate Casp2 [3]. At present, there are no reliable techniques for measuring Casp2 activity directly in human brain tissue. Therefore, we measured Casp2 levels as a proxy for Casp2 activity. To assess Casp2 levels we used western blotting to measure levels of the 48 kDa zymogen, and found ~ 59% higher levels of Casp2 in LBD than PD (Fig. 1d, e). Elevated levels of Casp2 are also observed in AD [11, 18], suggesting LBD and AD share a common pathway leading to increased levels of Casp2.

### Soluble total tau is lower in LBD than PD

To determine whether higher levels of Δtau314 in LBD were due to higher levels of soluble tau, we measured total tau (T-tau) by ELISA in the same aqueous extracts. Surprisingly, we found ~ 40% lower levels of T-tau in LBD (36.88; 27.32–58.1 μg/g) than PD (63.58; 59.54–96.48 μg/g) (Fig. 1b). The reduced levels of T-tau resulted in ~ 4-fold higher levels of Δtau314 normalized to T-tau in LBD (0.1248; 0.08135–0.2593) versus PD (0.02991; 0.01575–0.0684) (Fig. 1c). All comparisons remained significant after adjustment for age, sex, and PMI via multiple linear regression (Table 3).

### Neuronal marker β-III-tubulin is lower in LBD than PD

**Fig. 2:**
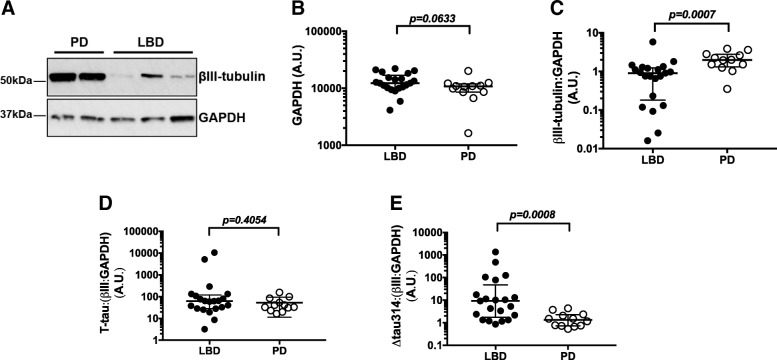
Levels of β-III-tubulin are lower in LBD than PD. **a** Representative western blots of β-III-tubulin and GAPDH in aqueous extracts. **b** No significant difference in GAPDH levels between LBD and PD. GAPDH was used as a loading control. **c** Quantification of β-III-tubulin normalized to GAPDH. Normalized β-III-tubulin levels are lower in LBD than PD. **d** T-tau normalized to normalized tubulin (β-III: GAPDH). No difference in normalized T-tau levels. **e** ∆tau314 normalized to normalized β-III-tubulin are higher in LBD than PD. Data were analyzed using Mann-Whitney tests. Bars indicate medians and interquartile ranges

To determine whether lower levels of T-tau in LBD was due to neuron loss or axonal degeneration, we analyzed β-III-tubulin in the same aqueous extracts by SDS-PAGE and western blotting (Fig. 2a). GAPDH levels did not differ between LBD and PD groups, and thus served as a loading control (Fig. 2b). We found a ~ 50% decrease in β-III- tubulin levels in LBD relative to PD, suggesting significant numbers of neurons or axons are lost in the superior temporal gyrus of LBD relative to PD patients (Fig. 2c). There was no difference in the T-tau: β-III-tubulin ratio between LBD and PD (Fig. 2d), indicating that the decrease in T-tau is attributable largely to neuron or axon loss. The Δtau314:(β-III-tubulin:GAPDH) ratio was ~ 7-fold higher in LBD than PD (Fig. 2e), reflecting disproportionately high levels of Δtau314 in the surviving neurons.

### APOE ε4 is associated with higher levels of Δtau314

Although APOE status is not associated with PD diagnosis [5], APOE ε4 is a genetic risk factor for LBD [8]. The ε4 allele of APOE is a strong risk factor for DLB, conferring an odds ratio of 2.4–6.1 [8, 21]. The APOE ε4 allele frequency is higher in PDD and DLB groups than in normal subjects or non-demented PD patients [14, 21]. Consistent with prior reports, in this study an APOE ε4 allele was present in 7 out of 21 LBD patients, but no PD patients.

**Fig. 3:**
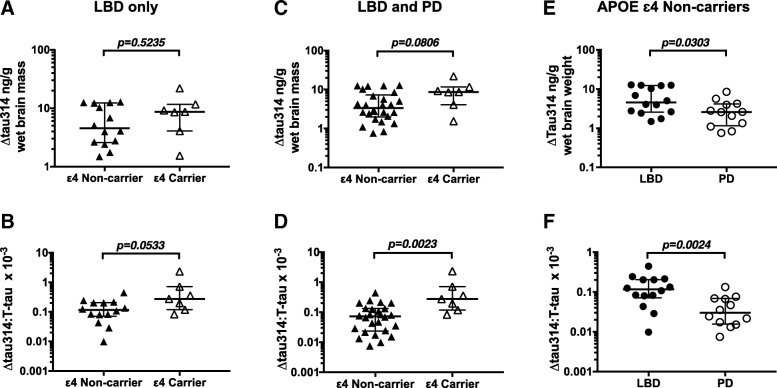
∆tau314 and ∆tau314: T-tau ratio levels in APOE ε4 carriers and non-carriers. **a**, **b** ∆tau314 and the ∆tau314: T-tau ratio in APOE ε4 carriers versus non-carriers in only patients with LBD. **c**, **d** ∆tau314 and the ∆tau314: T-tau ratio in APOE ε4 carriers versus non-carriers in subjects with Lewy body disease (LBD and PD subjects combined). The ∆tau314: T-tau ratio is higher, and ∆tau314 trends higher, in APOE ε4 carriers. **e**, **f** Δtau314 and ∆tau314: T-tau ratio APOE ε4 non-carriers. ∆tau314 and the ∆tau314: T-tau ratio are higher in LBD. Data were analyzed using Mann-Whitney tests. Bars indicate medians and interquartile ranges

To detect a possible association between Δtau314 and APOE, we compared Δtau314 levels in APOE ε4 carriers and non-carriers. We first examined the subjects with LBD, and found no significant differences in the levels of Δtau314 (Fig. 3a). However, we found an ~ 2-fold higher ratio of Δtau314: T-tau in APOE ε4 carriers (0.2751; 0.1178–0.7082; *n* = 7) compared to non-carriers (0.1162; 0.07089–0.205; *n* = 14) (Fig. 3b).

**Fig. 4:**
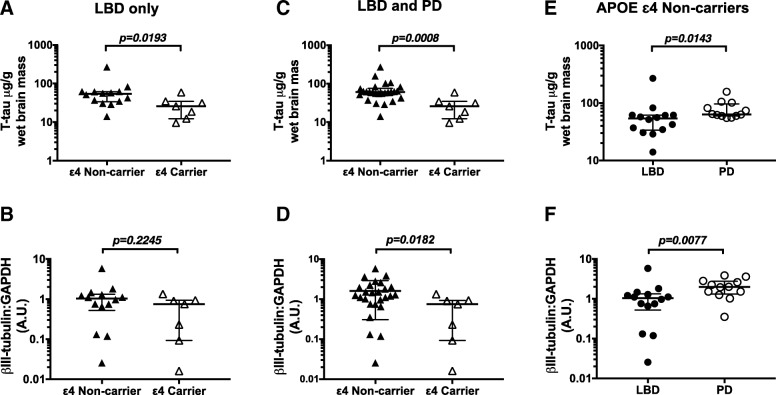
T-tau and β-III-tubulin levels in APOE ε4 carriers and non-carriers. **a**, **b** T-tau and β-III-tubulin levels in APOE ε4 carriers versus non-carriers in only patients with LBD. **c**, **d** T-tau and β-III-tubulin in APOE ε4 carriers versus non-carriers in subjects with Lewy body disease (LBD and PD subjects combined). T-tau and β-III-tubulin are lower in APOE ε4 carriers. **e**, **f** T-tau and β-III-tubulin in APOE ε4 non-carriers. T-tau and β-III-tubulin are lower in LBD. Data were analyzed using Mann-Whitney tests. Bars indicate medians and interquartile ranges

When we analyzed LBD and PD subjects together, we found a trend toward higher levels of Δtau314 in APOE ε4 carriers (8.667; 4.064 - 11.63 ng/g; *n* = 7) relative to non-carriers (3.376; 1.988–7.265 ng/g; *n* = 26) (Fig. 3c), and an ~ 4-fold higher Δtau314: T-tau ratio in APOE ε4 carriers (0.2751; 0.1178–0.7082) compared to non-carriers (0.0726; 0.02375–0.1328) (Fig. 3d). T-tau levels were lower in APOE ε4 carriers (Fig. 4a, c).

To determine whether Δtau314 associates with dementia independently of APOE ε4, we compared Δtau314 in demented (LDB) and cognitively intact (PD) APOE ε4 non-carriers. We found ~ 2-fold higher levels in LDB (4.55; 2.57–12.27 ng/g; *n* = 14) than PD (2.59; 1.16–4.16 ng/g; *n* = 12) (Fig. 3e, f), indicating that factors besides APOE ε4 status influence the association between Δtau314 and dementia in Lewy body disease. In APOE ε4 non-carriers, T-tau was 33% lower in LBD (49.7 μg/g wet brain weight) than PD (74.1 μg/g wet brain weight) patients. (Fig. 4e).

To establish whether neocortical neuronal or axonal loss is associated with APOE status, we compared β-III-tubulin levels in APOE ε4 carriers and non-carriers. There was no difference in β-III-tubulin (normalized to GAPDH) levels in LBD (Fig. 4b). However, we found β-III-tubulin levels (normalized to GAPDH) were 62% lower in APOE ε4 carriers than non-carriers when subjects were grouped together  (Fig. 4d). Among APOE ε4 non-carriers, β-III-tubulin levels in LBD were 40% lower than in PD (Fig. 4f), indicating that while APOE ε4 status may contribute to neuronal or axonal loss it is not the sole factor.

### Receiver operating characteristics of Δtau314, neurofibrillary tangles and Lewy bodies

**Fig. 5:**
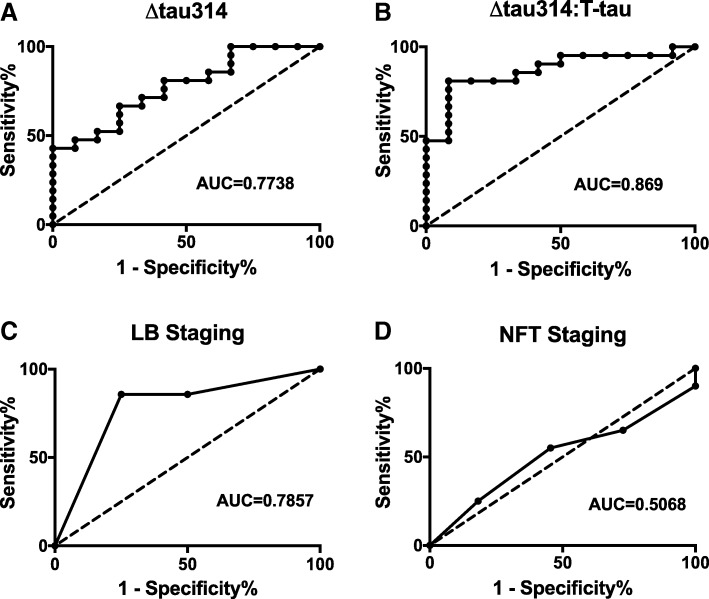
ROC analyses of ∆tau314, ∆tau314: T-tau, Lewy body staging, and neurofibrillary tangle staging in LBD versus PD. ROC curve analysis of **a** ∆tau314, **b** ∆tau314: T-tau, **c** Lewy body (LB) staging, and **d** neurofibrillary tangle (NFT) staging (using Braak criteria). The predictive value for dementia of ∆tau314, ∆tau314: T-tau and Lewy body staging are comparable, and superior to NFT staging. Dotted line is the line of identity (AUC = 0.5)

To assess the predicative value for dementia of Δtau314, we generated receiver operating characteristic (ROC) curves. The AUC was 0.7738 for Δtau314 (Fig. 5a) and 0.869 for the Δtau314: T-tau ratio (Fig. 5b). The AUCs for Δtau314: T-tau ratio (AUC = 0.869), Δtau314 (AUC = .7738) and Lewy body staging (AUC = 0.7857) did not differ significantly (Fig. 5c, Table 4). Braak staging, a measure of the distribution of neurofibrillary pathology, was not predictive (Fig. 5d, Table 4), consistent with the exclusion of subjects with AD pathology from the study cohort.

**Table 4:** Statistics for Fig. 3. Data were analyzed using Delong’s. Method for comparison of receiver operating characteristic curves

ROC comparison	*P*-value
△tau314 vs. △tau314:total tau	0.12
△tau314 vs. LB stages	0.92
△tau314 vs. NFT stages	0.11
△tau314:total tau vs. LB stages	0.45
△tau314:total tau vs. NFT stages	0.007

## Discussion

The soluble tau proteolytic fragment Δtau314 is formed specifically when Casp2 cleaves tau at Asp314, and is associated with the disruption of postsynaptic excitatory neurotransmission. Here, we measured Δtau314 in aqueous extracts of the superior temporal gyrus in subjects with Lewy body disease, and compared its levels with regard to the presence of dementia (i.e., Lewy body dementia, LBD) or the absence of dementia (i.e., Parkinson’s disease, PD). We found higher levels of Δtau314 in LBD than PD, and corresponding decreases in soluble T-tau and β-III-tubulin, a molecular proxy for neuron number or axon density. In addition, we found that Casp2 levels are elevated in LBD compared to PD; Casp2 levels in AD are higher than in controls, suggesting that AD and LBD share a common mechanism leading to increased Casp2 levels [11, 18]. These results suggest that in LBD there is pronounced cortical neuron loss or axonal degeneration with disproportionately high levels of Δtau314 in the remaining neurons. ROC analyses showed that Δtau314 performed as a predictor of dementia as effectively as Lewy body staging, the current gold standard.

We previously found increased levels of Δtau314 in the inferior temporal gyrus of subjects with MCI and AD compared to normal controls [23]. Recently, we found increased levels of Δtau314 in the prefrontal cortex and the caudate nucleus of patients with Huntington’s disease (HD) compared to control subjects (Liu et al., Acta Neuropathologica Communications, this issue). Taken together with the findings presented here, we speculate that in AD, HD, and Lewy body disease, cognition becomes impaired when Δtau314 accumulates in the neocortex. Our observation that Δtau314 and the Δtau314: T-tau ratio are equivalent to Lewy body staging for predicting dementia provides a strong rationale for attempting to measure Δtau314 in blood or CSF, and for determining its potential utility as a molecular biomarker of synaptic dysfunction in living subjects.

The association of LBD with elevated levels of Δtau314 is a novel finding. The importance of this result is that it lends support to the potential use of Casp2 inhibitors to ameliorate dementia in LBD. Several factors make Casp2 an attractive drug target. Not only does lowering Casp2 protect against several forms of neuronal insults, the absence of Casp2 has relatively few deleterious effects, at least in mice. The main adverse effects in Casp2 knockout mice are age-related bone and body fat loss and impaired hair growth [22]. Intraocular QPI-1007, an experimental siRNA against Casp2 used to treat non-arteritic anterior ischemic optic neuropathy, has thus far shown no serious harmful effects in clinical trials [1]. These features make targeting Casp2 a viable option for treating CNS diseases.

Casp2 has been implicated in mouse models of four neurodegenerative diseases —the rTg4510 model of frontotemporal dementia (FTD) [23], which expresses mutant human tau, the J20 mouse model of AD [15], which expresses mutant human APP, the YAC128 mouse model of HD [2], which expresses mutant human huntingtin, and the MPTP mouse model of Parkinson’s disease (PD) [19]. Lowering Casp2 using an antisense oligomer decreased Δtau314 production and improved memory function in rTg4510 mice, and prevented the mislocalization of tau to dendritic spines and reductions in excitatory postsynaptic neurotransmission [23]. Genetically ablating Casp2 in J20 mice reduced abnormal dendritic spine morphology and cognitive dysfunction [15], and conferred resistance to Aβ-mediated neurotoxicity in cultured neurons [20]. Knocking-out Casp2 in YAC128 mice ameliorated cognitive deficits, although neurodegeneration was not affected [2]. Casp2 knock-out mice resisted neurotoxicity induced by the nigrostriatal neurotoxin MPTP [19].

APOE status affected the levels of neocortical Δtau314, T-tau and β-III-tubulin. APOE ε4 carriers showed more profound neuron loss or axonal degeneration, but disproportionately higher levels of Δtau314 in the remaining neurons. APOE ε4 has been shown to increase tau-mediated neurodegeneration in P301S mice, and in several human tauopathies [17]. Interestingly, APOE ε4 can induce mitochondrial dysfunction and oxidative stress [4], and mitochondrial oxidative stress can, in turn, activate Casp2 [13]. Thus, it is possible that APOE ε4 may act in concert with mitochondria to increase Casp2 activity, leading to Δtau314 production and neurodegeneration. APOE ε4 is not exclusively responsible for neurodegeneration, however. Even in APOE ε4 non-carriers, LBD patients had higher levels of Δtau314 and lower levels of β-III-tubulin than PD patients, indicating that factors other than APOE also influence neuronal viability and Δtau314 production.

The main limitations of our study are the group sizes and the sampling of one brain region only. Group sizes ranged from 12 to 21, and only the superior temporal gyrus was examined. A study of different brain regions in a larger cohort is needed to confirm our results. It would also be interesting to test the idea that Δtau314 may correlate with the severity of motor signs in brainstem regions that are classically associated with Lewy body disease, such as the substantia nigra and striatum.

## Conclusion

In this study, we report the novel finding that the Casp2-generated tau fragment Δtau314 is associated with dementia in Lewy body diseases. Our results suggest a mechanism for the synaptic dysfunction underlying dementia in these disorders, and support the development of Casp2 inhibitors to treat Lewy body dementia.

## Data Availability

All data generated or analyzed during this study are included in this published article.

## Abbreviations

AD: Alzheimer’s disease
Casp2: Caspase-2
DLB: Dementia with Lewy bodies
FTD: Frontotemporal dementia
HD: Huntington’s disease
LBD: Lewy body with dementia
MCI: Mild cognitive impairment
OR: Odds ratio
PD: Parkinson’s disease
PDD: Parkinson’s disease with dementia
T-tau: Total tau
